# Aspirations for quality health care in Uganda: How do we get there?

**DOI:** 10.1186/1478-4491-11-13

**Published:** 2013-03-22

**Authors:** Clare I R Chandler, James Kizito, Lilian Taaka, Christine Nabirye, Miriam Kayendeke, Deborah DiLiberto, Sarah G Staedke

**Affiliations:** 1 Department of Global Health & Development, London School of Hygiene & Tropical Medicine, 15-17 Tavistock Place, London, WC1H 9SH, UK; 2 Infectious Diseases Research Collaboration, Mulago Hospital Complex, PO Box 7475, Kampala, Uganda; 3 Department of Clinical Research, London School of Hygiene & Tropical Medicine, Keppel St, London, WCIE 7HT, UK

**Keywords:** Africa, Access to health care, Power/empowerment, Quality of care, Relationships, Health care

## Abstract

**Background:**

Despite significant investments and reforms, health care remains poor for many in Africa. To design an intervention to improve access and quality of health care at health facilities in eastern Uganda, we aimed to understand local priorities for qualities in health care, and factors that enable or prevent these qualities from being enacted.

**Methods:**

In 2009 to 2010, we carried out 69 in-depth interviews and 6 focus group discussions with 65 health workers at 17 health facilities, and 10 focus group discussions with 113 community members in Tororo District, Uganda.

**Results:**

Health-care workers and seekers valued technical, interpersonal and resource qualities in their aspirations for health care. However, such qualities were frequently not enacted, and our analysis suggests that meeting aspirations required social and financial resources to negotiate various power structures.

**Conclusions:**

We argue that achieving aspirations for qualities valued in health care will require a genuine reorientation of focus by health workers and their managers toward patients, through renewed respect and support for these providers as professionals.

## Background

In spite of significant global investment, the majority of developing countries are not on target to achieve Millennium Development Goals 4 and 5, to reduce the under-5 mortality rate by two-thirds and the maternal mortality ratio by three-quarters between 1990 and 2015 1. Failure to reach these targets has been blamed on ‘health system bottlenecks’ that prevent the ability to scale up coverage of key interventions 1,2. Inadequate ‘building blocks of health systems,’ namely the numbers and distribution of health workers, equipment, supplies and infrastructure, are cited as contributing to low coverage of health interventions, with median rates of correct treatment of childhood diarrhoea, pneumonia and malaria below 50% 3. Accelerated efforts to meet the 2015 targets focus on ‘evidence based interventions’ to be supported by ‘strengthened health systems’ (*ibid*.). However, many argue that the way services and programs are enacted in practice is a social as well as a structural issue: a function of interactions between clients, communities, health workers and systems 4,5.

Meeting a population’s expectations from provider services has been recognised as central to health system performance 6. The importance of meeting health worker needs in order to deliver good quality, patient-oriented services has also been recognized 7,8. However, many interventions continue to take a ‘magic bullet’ format, with limited effects. Interventions such as user fees for patients have not been shown to improve access or health outcomes, or to decrease health expenditure 9, and skills training and guideline changes aimed at health workers have had limited impacts on changing practices 10,11. Likewise, investment in human or equipment resources in efforts to strengthen health systems have had limited effect in the absence of efforts to improve health-service management and coordination 2.

Uganda has seen the implementation of many programs intended to improve health and access to health care since the early 1990s. However, health, and access to health care remain poor across the country 12. Social studies of the introduction of these interventions suggest that their limited effects may be attributable to a lack of alignment between programme priorities, defined externally to the local population, and local priorities 13,14,15,16,17. The design of health programs does not appear to take these local realities into account. Understanding local aspirations and the moral landscapes into which programs are introduced and enacted may provide insights for more nuanced approaches to improving health care 18. Intervention design built upon the lived realities of those enacting health care differs from regular programmatic approaches, but may provide alternative ways to improve health through better services in rural Uganda and in other areas of Africa.

We undertook a qualitative study, alongside a large census and health-provider survey in 2009 to 2010, to understand 1) priorities for quality in health care from the perspectives of health workers and community members in Tororo District, Uganda and 2) factors potentially amenable to change that could enable these qualities to be enacted, thereby increasing equitable access to good quality health care for the local population. Our focus was particularly on health care for children, as a key vulnerable group. This qualitative research informed the design of a complex health-facility intervention that is under evaluation as a large cluster randomised trial in Tororo District from 2011 to 2013 (clinicaltrials.gov NCT01024426).

### Theoretical orientation

Anthropological studies have repeatedly emphasized the importance of social relationships in the enactment of health care 19,20,21, and have drawn attention to how these social and cultural realities are embedded in particular political-economic and historical contexts 22. In this article we analyse the lived realities of the enactment of health care, as expressed by community and health-worker participants, in the context of the local political history of the study area. We use the term ‘enactment’ to describe the moments when health care is produced, rather than the terms ‘access’ or ‘service provision’, which evoke concepts such as ‘utilization’ and ‘availability,’ based on numbers and structures. Studies of access to health care in practice suggest its enactment is a dynamic interaction between populations and services, and health seekers and health workers, over extended time periods, often in contexts of social and economic as well as health vulnerability 5,16,23,24,25,26. In this article, we attempt to draw together meanings and practices of care as interpreted and experienced by actors who seek and provide health services.

### Study setting

#### Uganda: health system changes

During the economic deterioration of the 1970s and 1980s, when the weakened Ugandan state struggled to provide free health services and to pay for health-worker salaries and the upkeep of facilities 27, health workers were observed to develop ‘survival strategies’ to cope with scarce resources, including adopting external profit-making practices 28, and the use of public resources in their private practices 29.

To improve accountability at the local level, health care in Uganda was decentralized in 1993. Local authorities were handed responsibility for health-care activities outside of hospitals. This included responsibility and authority to set fees for services provided, which could feed into district or facility funds. Contrary to hopes that introducing fees would formalize payments, the ability to charge for services, together with continued low salaries, resulted in increased leakage of drugs 29, mismanagement of funds 30, informal requests for payment from patients, and reduced quality and accessibility of care 31. Health workers were continuing to rely on their ‘survival strategies’ implemented in earlier years 15. The shift in power to charge fees was mirrored by a shift in power to recruit staff and allocate resources at the local level, which was intended to empower responsiveness to local needs. In practice, however, the ability of local politicians to define local needs created friction within districts. Health workers needed to reinforce their relationships with leaders who interpreted decentralized policies as preferences for locally born staff 32. Selection criteria for training opportunities were not based on training needs, with in-charges attending more seminars and reaping the benefits 15. With competition for income opportunities, health workers reportedly prioritized attendance at meetings for which allowances would be paid over providing care to patients (*ibid*.).

In 2001, Uganda’s President, Yoweri Museveni, abolished user fees as part of his election campaign. The health sector strategic plan for the turn of the millennium identified the health sector as playing a key role in poverty eradication and socioeconomic development in the country 33. Analyses of the removal of user fees in Uganda suggest a positive impact; quality of care did not decrease 34, and more poor people sought care at public facilities. However, the proportion of poor households facing catastrophic health expenditures did not decrease 35. For health workers, removal of user fees meant a sudden influx of patients to health centres, stretching supplies, staff and space 36.

#### Tororo: interventions and health

The significant changes in the organization and delivery of health care in Tororo District over the past 20 years reflect both the changes on-going in Uganda nationally and various donor activities in the district. Tororo was one of the first districts in Uganda to be decentralized, in 1994, with implications similar to those described elsewhere in the country 17. Subsequent upgrading of health facilities to the vision of the Health Sector Strategic Plan 33, including a referral level health centre (Health Centre-HC-IV) per county, a mid-level HCIII per sub-county and a low-level HCII per parish, was slow to be implemented. In 2003, Tororo District had only 50% of the desired facilities 37, with a staffing gap of 73% 17. In 2009, our survey in one sub-district of Tororo found that while higher level facilities were in place, only 56% of the parishes had health facilities and there was a 41% staffing gap of those officially in post 38when compared with staffing norms set out in the 2005 Health Sector Strategic Plan 39. Donors have contributed significantly to district funds, through both a sector-wide approach and direct support to a multitude of programs, particularly to HIV/AIDS care and prevention and malaria prevention and control 40.

In spite of these efforts, data relating to health and wealth of citizens of Tororo in 1996 and 2003 suggest no significant improvement in socioeconomic status of households overall, and some worsening of childhood illnesses including diarrhoea, fever and acute respiratory infections 40,41. Our census survey in 2009 to 2010 showed the area continued to be poor, with few households having electricity (1%) and most obtaining water from a public borehole or well/spring. A quarter of households had no toilet facilities. Of the heads of households, 24% had received no formal education. Mortality in children under 5 years of age was estimated at 11% 38. A survey among patients and community members in 2003, at a time when drug availability was reported to be strong, suggested quality of services was perceived to be generally good although dissatisfaction was expressed with waiting times, staff availability, some rude staff, language difficulties and having to pay for treatment 17. In both 2003 and 2005, health-care workers complained of staff shortages; limitations in training opportunities, working equipment, drug supplies, and working space; and difficult relationships with politicians 17,36. Both surveys recommended the increase and use of funds to fill gaps in staffing, equipment, supplies and technical skills.

#### West Budama North: health facilities

We carried out this qualitative study in the West Budama North sub-district of Tororo, where the population is largely of the Japadhola ethnic group. Seventeen government-run health centres were operational in the study area, including 12 health centre (HC) IIs, the lowest level where medicines are dispensed, four HCIIIs, where patients may stay overnight and babies are delivered, and one HCIV, which provides referral care and has several wards. HCIIs and HCIIIs reported seeing between 50 and 60 patients per day, and the HCIV around 200. HCIIs were staffed by one to three health workers, while HCIIIs had approximately ten, and the HCIV had 36 health workers, although only around half of the health workers stationed at these higher level facilities were available to work on a given day. Shortages in drugs and equipment were noted at all HCs and many lacked running water and electricity. Of the health workers at facilities visited in the survey, 26% were volunteers with no official post, and frequently no qualifications, but whose roles included dispensing medicines, giving immunizations and even delivering babies. The study area also housed a number of private drug shops, also known as ‘clinics’, usually owned by health workers.

## Methods

After an intensive training course in the study’s objectives and in methods, led by CC and based on a manual for Quality Information in Field Research 42, a team of six social scientists carried out fieldwork in Tororo District from September 2009 to March 2010. Activities specific to the research question presented here consisted of a series of in-depth interviews followed by focus group discussions (FGDs) with health workers and a series of FGDs with community members. The social scientists were based in a rural town for the duration of fieldwork, enabling a richer understanding of the local political, economic and health context. Three of the team were from the local area and able to speak Japadhola while the remaining team members were from other areas of Uganda and carried out fieldwork in Luganda or English.

### Study sample

Community focus group discussions were held with primary care givers of children under 5 years of age, as the most frequent users of primary care services, and with heads of households, deemed important for their influential role in accessing health care. We used a sampling matrix (Table 1) to allocate different subgroups of interest among ten FGDs, stratified by each of the five sub-counties in the study area, communities that had a health facility within and outside of their parish, age group of primary caregivers, and gender of household heads. Further FGDs would have been conducted if new themes continued to emerge in any sub-group.

**Table 1:** Sampling matrix for community focus group discussions

**Primary Caregivers**					**Heads of Household**				
**<30 years**		**>30 years**			**Female**		**Male**		
SC1	SC2	SC3	SC4	SC5	SC1	SC2	SC3	SC4	SC5
PHC	PHNC	PHC	PHNC	PHC	PHNC	PHC	PHNC	PHC	PHNC
FGD201	FGD202	FGD203	FGD204	FGD205	FGD206	FGD207	FGD208	FGD209	FGD210

SC, sub-county, with numbers randomly allocated to sub-counties in West Budama North;

PHC, parishes with health centres; PNHC, parishes with no health centres;

FGD, Focus Group Discussion study identification number.

All health workers at health facilities in the study area were invited to participate in an in-depth interview, including volunteers. All health workers were also eligible for participation in subsequent FGDs. To facilitate open discussion, FGDs were held separately for the three levels of health facility, and within these, separate FGDs were held for those with higher health qualifications, such as nursing officers and clinical officers, and for those with lower or no health qualifications, such as nurse assistants or volunteers. For the lowest level health centres, we invited those ‘in-charge’ of their health centres to a separate FGD.

### Participant invitation

Community leaders were informed about the study and were asked for permission to carry out the fieldwork. They were then asked to work with the study team to identify 8 to 12 participants to represent the subgroups of primary care givers and household heads from their communities. Participants were invited to attend the discussion at a local community hall or school classroom and refreshments and a transport refund were provided. Those in charge of health facilities were asked for permission to carry out the study and to provide a list of health workers posted to the facility. Health workers were invited to participate in an in-depth interview at their convenience and were contacted again to participate in a focus group.

### Focus group discussions and in-depth interviews

Participants were provided with an information sheet, which was read and discussed with a member of the study team. Participants who agreed to take part and to be audio recorded were asked to provide witnessed verbal consent. For the FGDs, each participant was given an identification badge with a number for anonymity and rules for confidentiality were discussed with each group. The FGDs and interviews then followed a topic guide, facilitated by a member of the study team in English, Japadhola or Luganda. The community participants were asked to discuss their experiences with illnesses and treatment seeking for their children, their perceptions of different providers and for suggestions on how to improve the delivery of care and appeal of public health facilities. Health workers were asked to discuss definitions of quality of care, opinions about their own delivery of care, relationships with patients and colleagues, and suggestions for improving quality of care and appealing to the community. Extensive notes and a contact summary were completed for each FGD and interview, shared and discussed in real time with the broader study team. Audio recordings were transcribed and translated and field notes were integrated. Each transcription and translation was cross-checked for accuracy and then imported, together with participant demographic information, into NVivo version 8 (QSR International, http://www.qsrinternational.com) for coding and analysis.

### Analysis

Analysis was on-going during and after fieldwork. This enabled us to incorporate issues arising, such as payment for services, into subsequent interviews and FGDs. Coding involved labelling ideas in transcripts and organizing these under headings that represented meanings underlying groups of ideas. After coding the first few transcripts from each participant group, the team agreed on a working template in which to code subsequent transcripts. Two members of the team undertook coding of the community transcripts and three the health worker transcripts. Each coded into separate NVivo files, which were merged frequently and the coding templates updated by CC, to reflect new ideas and themes emerging. Higher level analysis, linking themes together into broader concepts, evolved through a series of whole team discussions about the data and consultation of literature and theory.

### Ethics

The study was approved by the Ugandan National Council for Science and Technology (HS 644), the Makerere University Faculty of Medicine Research and Ethical Committee (2009–149) and the London School of Hygiene and Tropical Medicine Ethics Committee (5591).

## Results

We found that health-care workers and seekers valued technical, interpersonal and resource qualities in their aspirations for health care. However, such qualities were frequently not enacted, and our analysis suggests that meeting aspirations required social and financial resources to negotiate various power structures. In order to build on existing literature, which is extensive in describing priorities in health care qualities, we provide only a short summary of the qualities valued by participants and then focus on the latter issues of the enactment of care.

### Participants

In all, 69 health workers were interviewed, and 65 took part in 6 FGDs (Table 2). The mean age of health workers was 37.5 years, with a median of 5 years’ experience as a health worker. Over half were female and most who worked at lower level health centres were originally from the area. By contrast, most health workers from higher level health centres originated from outside of the area. A majority of health workers had at least a certificate level of education, with the most qualified working at the highest level health centre. However, 17.5% of those working at health centres had no health qualification, a majority of whom were volunteers.

**Table 2:** Demographic details of health worker participants at each health centre level

	**Total (N = 69)**
Age (years; mean, sd)	37.5 (10.8)
Gender (number of females, percent)	41 (59)
From the area (number, percent)	43 (62)
Years worked as a health worker (years; median, IQR)	5 (0.1 to 37)
Position at health centre (number, percent)	
In-charge	15 (22)
Nurse/midwife	15 (22)
Other trained health worker	9 (13)
Nursing assistant	11 (16)
Volunteer	19 (27)
Highest level of education (number, percent)	
Primary	1 (1.5)
Secondary	11 (16)
Certificate	45 (65)
Diploma/Bachelor’s	12 (17.5)

IQR, interquartile range.

A total of 113 community members took part in 10 FGDs (Table 3). Primary caregivers were younger than heads of household with a mean age of 33.8 and. 45.9 years, respectively. All primary caregivers were female, while 41% of the household heads were female, purposively selected for two of the FGDs. Participants in the two subgroups had similar numbers of children - a mean of 4.4. Participants were generally not well-educated; 30% (mostly women) lacked any formal education, and only 22% had attended any secondary school, all of whom were male.

**Table 3:** Demographic characteristics of community participants

	**Primary caregivers (n = 55)**	**Heads of households (n = 58)**	**Total (N = 113)**
Number of participants in a group (range)	11 (10 to 12)	12 (11 to 12)	11 (10 to 12)
Mean age (range)	33.8 (18 to 55)	45.9 (20 to 80)	40 (18 to 80)
Number female (%)	55 (100%)	24 (41%)	79 (70%)
Mean number of children (range)	4.8 (1 to 11)	4.1 (0 to 10)	4.4 (0 to 11)
Number with no formal education (%)	14 (25%)	20 (34%)	34 (30%)
Number with any primary education (%)	35 (64%)	19 (33%)	54 (48%)
Number with any secondary education (%)	6 (11%)	19 (33%)	25 (22%)

### Valued qualities in health care

Both community and health-care worker respondents conceptualized access to quality health care as a comprehensive therapeutic process: a compound of technical, interpersonal and resource factors. Respondents identified room for improvement on all fronts. Tables 4 and 5 summarize the qualities valued by health workers and patients in our study sample.

**Table 4:** Qualities valued in health care by health worker respondents

**Quality**	**Number of health workers saying they valued this quality in health care (of 69 interviews)**
Clinical care and treatment	34
Availability of drugs, staff, equipment and infrastructure	22
Good interpersonal interactions with patients	21
Giving advice	20
Welcome and guidance for the patient	12
Being professional	11

**Table 5:** Qualities valued in health care by community respondents

**Quality**	**Number of community focus groups saying they valued this quality in health care (of 10 focus group discussions)**
Free and timely services at the health centre	10
Good interpersonal interactions with health workers	10
Good management of health centre and resources	8
Good treatment provided by health workers	8
Good consultations, including asking questions, examining and testing patients	8
Welcome and orientation of patients	7
Advice and explanations given to patients	7

#### Technical quality of care

An important quality in health care described by both health workers and community respondents was good clinical care and treatment, entailing examination, investigation and diagnosis, followed by giving the right treatment to patients. We noted that such technical qualities were most often listed among other services valued in health care, as exemplified by this health worker,

Good quality health care. It’s receiving patients in a humble manner and giving them the correct treatment at the right time and a right diagnosis. Even counseling them and advising them. (Nursing aide at HC06, interview #41).

#### Interpersonal quality of care

Both health workers and community members valued interpersonal qualities highly in the delivery of good health care. These extended beyond providing advice or education to patients to the attitudes conveyed through receiving and welcoming patients, giving explanations and expressing concern and reassurance.

You first welcome, that one will make that person free and will make her air out her problems that she may be in need of telling you. There is also introducing myself to the patient, greeting the patient, then later on you can ask the patients what her problems are and attend to with keen interest, not just when the mind is very far . . . the focus should be on the client, and the mother or the client may know that you have been attending to her or her problems. (Midwife at HC15, interview #81)

The *musawo* [health worker] working there should welcome you, ask you questions very well. And you also explain to them. You know there are eyes [looks] that also scare. Then your heart beats. Then you fail to say what you wanted to say. You know there is a way we keep ourselves: you know someone who is educated and the one that is not. There is a way he/she can take you. So you will just sit there like a stupid person. The child is breathing badly from your hands but you are just there like a stupid person, very useless, surely when you know nothing. Even those things written on the door you do not even know where to enter. But if it were a kind *musawo*, who God created with kindness and was raised up well, reaches and sees that who is this who has reached, welcomes you well, ask you something carefully. Should teach you well, slowly, ask you while talking slowly. That is when you will remember everything. (Primary care giver, respondent 9 in FGD #209)

In spite of united aspirations for good quality care to incorporate good interpersonal skills and humility, the experiences of many community participants suggests that these aspirations were often not achieved in practice, discouraging patients from attending health centres. Community respondents felt that reform in health worker attitudes was urgently needed.

There are some health workers who have bad manners, they even want to beat you with your child, when a child is very sick, you have gone to call them but again they want to beat you, chase or send you away, sometimes [you] may even fall. So we were requesting that such habits should stop, we should be handled with good manners. (Female household head, respondent 8 in FGD #204)

#### Resources and quality of care

An essential quality in good health care was availability of resources, particularly of drugs and other equipment such as syringes and gloves, as well as availability of human resources to provide swift treatment. Lack of these resources, or charges made for access to resources, was also considered to undermine technical and interpersonal aspects of care.

I think good quality health care is when we have all the required logistics in place . . . in terms of medicines in the health facilities and the lab should have whatever is needed there, and things like injections should all be in place, the medical staff must be there ready to serve the community. But you realize that Tororo District is operating with about 47% of the staff required here. So that means that we are understaffed, and yet the influx of the community is great, who come for treatment and even the drug supply is seasonal. They have brought today but after one week there will be no drugs. (Health inspector at HC01, interview #08)

I also have a problem with that health centre. That place is the only health centre that actually represents the whole sub-county. But that place operates; I don’t know whether I should say that it operates for half a day, or for a quarter day. Because when they [are supposed to] start at 8 am, by 1 pm, there is nobody there, like a health worker. (Male household head, respondent 7 in FGD #206)

A district distributed ‘primary health care fund’ (PHC) is intended to pay for everyday supplies such as cleaning materials, or for transport to fetch drugs, or to make photocopies for reports to the district, or to pay for people to clear bushes or to clean toilets at health centres. However, the PHC fund had not been received by health centres in the study for at least 6 months, with ramifications for health centre infrastructure and staff morale,

Now like nowadays we don’t have PHC, the compounds are very bushy, which means you have to pull your own money to give the porter [casual labourer] . . . the recording book is finished is there, you have nowhere to write, you have to pull money and buy. Sometimes the nurse is rude [group laughs] but there are so many reasons why the nurse is rude. (In-charge of an HCII, respondent 2 in FGD #104)

All community FGDs included unprompted discussions about requests for payment for services that are officially free, reporting serious results for the poorest who were unable to pay, and discouragement of many from seeking care at health centres.

For me when I reach there [at the health centre], it is the money issue that scares me, because they will need money from me when I don’t have, yet my child is badly off, may be requires putting on drip, but there is no money. So instead they will chase me with a sick child that I go and look for money, now for me, I am a poor person, I just have to first dig somebody’s garden, before getting money and this then means that the child will have died, so that money issue is what I don’t want. (Primary care giver respondent 7 in FGD #201)

When health workers were asked during FGDs about payments from patients, some stated that this did not happen, but that patients were told to buy supplies from drug shops or pharmacies when the facility’s supplies were finished. However, others did inform us that they charged patients for services. Volunteers reported that such charges, and sales of record books and other commodities including syringes and gloves on site provided much needed income. These volunteer workers are unofficial and unpaid but their presence was ubiquitous at health facilities in this study, and official health workers reported they were an important human resource.

### Negotiating positions of power in the enactment of health care

Our analysis suggests that social relationships are at the heart of the enactment of good quality health care. Restrictions can be noted in absolute numbers of resources, and health worker abilities to provide technically and interpersonally good quality health care. Beyond these absolute restrictions, our analysis suggests a more dynamic situation in which managers, health workers and health seekers make decisions about when to use different resources and skills, related to their negotiation of positions of power. This can be exemplified through examples at health centres and in the wider district.

#### Negotiating power in health centres

Two major power differentials were recognized by participants in the health centre arena: between health workers and patients, and between higher level cadre health workers and those with lower or no qualifications.

Health workers described how they exercise their power to refuse or provide substandard or rude care to certain patient groups, particularly patients who they considered not to have any monetary or social capital to offer in return for services. This was most starkly exemplified by health workers who undertook spontaneous role-plays to demonstrate to us the differences between how they treated patients of different socioeconomic backgrounds, admitting that a better off client would be warmly welcomed with a ‘good smile,’ leaving other activities to offer services, whereas an untidy-looking, poor client would likely have to sit and wait, be criticized for attending in that state, spoken to with ‘scaring words’ and told ‘I can’t touch you when you are dirty.’

Community member narratives included many examples of this power at play, experiencing that waiting times, the level of interest expressed by the health worker for their concerns, and distribution of resources depended on a care seeker’s alignment with health centre culture, identified through their dress, manner, language and ethnicity.

There are those [health workers] who just look at you. When you arrive, they just continue conversing with their friends as though they have not seen you the patient.

She will just sit there and continue speaking her English. For you, you will sit there and she will help her friend whom she knows, who will come from the other side [end of the queue]. . . . She skips you and yet the child is breathing so badly and then she will attend to other person while for you, you are just there. (Primary care giver, respondent 5 in FGD #209)

Knowing how to present oneself at a health centre was seen as a great advantage in gaining access to quality services. Showing ability to pay for services was one strategy used, but beyond this, community members described portraying themselves in a particular way during a visit, even showing a status of power above the health worker, as well as making efforts to become familiar with health workers outside of the health centre.

For me when I reach [the health centre], I use any trick until I see that I talk well or speak with him well . . . there is a way I can come and talk to that person like someone who knows him. Like knowing his tribe, something like that. (Male household head, respondent 1 in FGD #210)

Maybe if he asks me where I come from as they do now days, I can deceive that I come from Gulu, that way, or even from the barracks [All laugh]. They will develop some fear, that ‘maybe this person has come to spy on us, so let us give him drugs as required.’ I have done this before and got better services. (Male household head, respondent 8 in FGD #208)

In spite of using these strategies to negotiate power and gain access to care, community members felt that it should not be necessary to resort to this. They expressed a desire that health workers provide equitable services routinely. This was often expressed in terms of wanting health workers to recognize that they as patients are human, and this should mean giving help when one is in need.

For me, I was thinking that these health workers are people, like us. They should see what happens to them, maybe if a relative is sick or has died. Normally when you have a patient or lose one, we need encouraging words. So they were supposed to work with good hearts, knowing that the government sends these services to everyone, and pays them [the health workers] (Male household head, respondent 1 in FGD #206)

Power differentials between health workers of different cadres also contributed to how health care was enacted. For example, seeking informal payments from patients or work-related opportunities was attempted by all levels of health worker, but mechanisms for doing so were more straightforward for those in positions of power, ‘big people,’ who were reported to seek opportunities to the disadvantage of their lower level colleagues through use of their positions of power.

But basically, sister, you being a person, a volunteer here and the in-charge or the big person has said ‘no selling of anything within here’ and you are caught, you are a volunteer and you will not be transferred anywhere else. . . . You have to go and tell your friend that ‘you go and give five hundred [to the health worker, he] will just give you treatment.’ So being a subject here you have to abide by the law, or the in-charge, because he is the one to ask for money [others laugh]! (Volunteer, respondent 5 in FGD #106)

The power of in-charges to decide who among staff can receive benefits was also noted by respondents, who reported biased choices based on favouritism or ‘tribalism’, with results of resentment among colleagues.

Like I had told you about workshops, you find that they select only one person. Like if someone has been trained on malaria, if there happens to be another workshop, still they send the same person and yet there are other people who are equally bright but they don’t know any knowledge about malaria. So because they know that there is money, they always send that person to benefit. And actually there is tribalism, like now, for us who are from very far away, with this system of decentralization, they consider their home girls and boys. Then for us who are from very far, you find that you are left behind all the time. . . . So there is that tribalism and segregation. (Enrolled nurse in HC01, interview #20)

The consequences of the observations of power abuses by senior colleagues led to the need to instigate alternative ‘survival strategies,’ especially for those with little or no salary. This inevitably compromised the quality of care they provided to patients, who were targeted as sources of income rather than seen as in need of care. Volunteers reported that they might charge around 500 shillings (approximately 0.25 USD at the time of the study) for helping patients (for example those in need of maternity care or for dressing dirty wounds):

A patient who was having wounds, dirty wounds, so maybe you ask something from him, or I don’t know, otherwise some also demand some money, for maybe dressing the wound . . . for the service rendered. (Volunteer, respondent 4 in FGD #106)

#### Negotiating power in the district

We observed that the quality with which health care was enacted was also dependent upon the ways power was negotiated within districts. Here we describe this in terms of relationships between district officials and health centre staff, although we also noted the importance of negotiating power within households for accessing care, which is outside of the focus of this analysis.

Power negotiations were evident in the chronic problems of drug stock outages at the time of the study, where local political campaigns pointed blame at health workers for stealing drugs. Health workers in this study reported feeling powerless in the face of these critiques - unable to acquire the drugs they needed through requisitions to the district and reluctant to suggest that patients purchase drugs from pharmacies for fear of being reported as selling ‘stolen’ drugs.

Those politicians just stand there and say, ‘Drugs have been taken to the health unit and the nurses are stealing them,’ which is not true. It may be happening in certain places but at least they must watch and see. It is not in every place. (In-charge of a HCII, respondent 2 in FGD #104)

In most cases you find that the political will is not working hand in hand with the health workers and instead they point fingers, they blame and they don’t bother about the welfare of the health workers. (High cadre health worker, respondent 12 in FGD #103)

Health workers also spoke of having to negotiate power held by district level health officials in accessing other resources, including PHC funds for health centres and salaries for official staff, which respondents had experienced being withdrawn for political reasons. Health workers described taking opportunities of supervision visits from the district to discuss these challenges for their health centres, but these visits were scarce and seldom resulted in hoped for results. This reinforced health workers’ feelings of a lack of control in contrast with their district superiors.

Sometimes we don’t get feedback from the district or Ministry of Health. We send reports every month, they come to supervise us and get problems from here and take back there but at times, those problems are not solved. . . . What I can say is that in most cases, you find that those who come to supervise are after fault finding, so such people may not be beneficial to us. (In-charge at HC13, interview #71)

You find you have a problem and you go [to the district] and explain to the right person, the person is just swinging as if it’s not a problem, something small. Just because for him he gets his salary, [he] will not mind much. So you will not have any motivation to work there [at the health centre]. (High cadre health worker respondent 1 in FGD #103)

Health centre staff were keen to enter into dialogue with district staff, if a discussion could be two way. They were especially keen for high level officials to see what it is like to work and be treated in their health centres.

We should also have a regular discussion like this one. We take long without having [such]. In fact it has not been happening. If higher ranking officers can do something like that, because we are just down there crying and they are just there being bosses. They don’t know what is happening. (In-charge of a HCII, respondent 9 in FGD #104)

In summary, although health workers and community members shared a similar view on the qualities desired in health care, the enactment of these qualities depended upon social relationships, and in particular, the navigation of power when differentials were steep.

## Discussion

Improving access to good health care is considered central to achieving health goals 43. In Uganda, as in many countries, interventions to improve access to health care have focused on structural components of health systems: intermittent investment in facilities, training of (some) health workers and changes in fees for services. So far, these interventions have had limited effect on health in our study area. We found that valued qualities in health care went beyond absolute resources and skills, particularly to positive interpersonal interactions, and that the enactment of these valued qualities was contingent on navigation of power relationships by different actors including health care seekers, health workers and district officials. Those in, or able to imitate, a stronger social position, and who developed effective strategies to navigate power, were able to fulfil their immediate objectives most effectively. We argue that achieving aspirations for qualities valued in health care will require a genuine reorientation of focus by health workers and their managers towards patients, through renewed respect and support for these providers as professionals.

The qualities valued in health care reported by health workers and community members in this study, including the emphasis on interpersonal qualities, echo the findings of others in Uganda 16,44,45 and elsewhere in Africa 46,47,48. We particularly noted the significance of a good ‘welcome’ to both health care seekers and workers. In spite of a wealth of evidence that interpersonal qualities are important in patient choices of health care 49, with consequences for uptake of services 50,51 and health outcomes 52, relatively few programs in low-resource settings systematically address these qualities. This may in part reflect the priorities entrenched in the ‘biomedical model’ of the centrality of technologies and technical skills for identifying standard disease entities as opposed to ‘patient-centered’ models which stress the individuality of patients and providers, necessitating interpersonal qualities for successful health care 53. With limited resources, a focus on interpersonal aspects of care may seem like ‘icing on the cake.’ It may also reflect a recognition of the difficulty of changing the way individuals behave towards each other, understood to be part of ‘social context,’ often conceptualized as a ‘factor’ that is difficult to change 54. Our analysis suggests that, rather than the social world being a ‘contextual’ factor, or a factor only affecting interpersonal aspects of care, all qualities valued in health care in this setting are embedded in social relationships, which shape how these qualities are enacted. Thus the social world is the medium through which care is acquired and provided, and also the potential medium through which to enable aspirations for qualities to be met.

### Implications for programs

We suggest that changing the way that health care is enacted, to realize the aspirations of both seekers and providers of health care, will require a reconceptualization of services and the different actors who bring these to life. In their comparison of the organizational models of European and African health care systems, Blaise and Kegels 55 note a legacy of ‘extreme standardization and rigidity of hierarchical command and control systems’ in Africa. They caution against interventions that reinforce standardization and external control, and argue instead that instilling professionalism may promote ‘more flexibility, patient-focus and responsiveness’. Their caution is supported by evidence that guideline and skills-oriented interventions have limited effects on quality of care 11,56 and observations that peers and perceptions of position among a wider community of practice play a comparatively more important role in practice 57. However, it has been argued that conventional health system development strategies in Africa continue to undermine local agency and contribute to the disempowerment of health workers, managers and policy-makers at all levels, who feel unable to effect changes that may improve the quality and impact of services 58. The professionalization of health workers, particularly of mid-level cadres, whose jobs, resources and reputations are subject to those in higher positions of power, could lead to a greater confidence, self-esteem and value 58. It is likely that such attempts will be most successful if basic monetary needs of health workers are met first 59. In turn, better motivated health workers may have more inclination to deliver better care to patients 7.

Facilitating such shifts in conceptualizations of health workers, and in turn of patients, will be challenging. The mode of service expected and promoted by health programs is situated within powerful discourses emanating from the fields of development and of biomedicine. Possibilities for change on the local level may therefore be limited, although not impossible, especially for decentralized systems. Communities of practice, in which those engaging frequently with each other shape norms and expectations, may be loci for such change 60, as has been observed in malaria case management in Ghana 61. Annemarie Mol suggests that a medium for making improvements in health care is through sharing stories and airing mistakes and uncertainties, in an environment where individuals are not on the defensive, noting that currently there is often ‘no room for doubt, self-criticism, or difficult questions. However, improvement begins with the recognition that something needs to be improved.’ 19. Furthermore, she observes that, ‘rather than a matter of ‘merely’ sharing private experiences, telling stories is a form of public coordination. It is part of how we govern ourselves and each other.’ (*ibid*.). Such story-telling could be incorporated into approaches that have shown some success in improving patient-centeredness through building self-awareness by facilitated reflection and meditation in Africa 62 and elsewhere 63.

Building on the findings of the research presented in this paper, we have designed an intervention based on the qualities for health care aspired to by participants. This consists of a series of workshops and self-observation activities to stimulate patient-centered services alongside training and tools for the management of supplies and funds at health centres and training and supervision in malaria case management including new rapid diagnostic tests. The intervention also provides malaria-related supplies to the health sub-district level, to be requisitioned from health centres through a specific process. This will be evaluated through a cluster randomized trial accompanied by a comprehensive process, context and impact evaluation (clinicaltrials.gov NCT01024426).

### Limitations

A total of 182 health workers and community members participated, and we are unable to represent the breadth and depth of each of their views here. However, in this article we aimed to present a more nuanced analysis of the way health care is enacted in this district of Uganda. Our interpretation draws on our understanding of participants’ words and meanings in the context of our theoretical orientation, outlined above. We suggest that our approach of working closely as a team throughout data collection and analysis and challenging our own thinking and assumptions throughout strengthened the validity of our interpretation. The generalizability of the findings may be limited in the specifics but we believe that by drawing on our empirical data together with insights from other authors, the concepts may be transferable to different settings.

## Conclusions

In spite of decades of reforms and interventions to improve health through access to quality health services, these goals remain unmet in eastern Uganda. In contrast to the focus of programs on absolute resources and skills, health-care seekers and providers in this study had unified aspirations for qualities of health care as a compound of technical, interpersonal and resource factors. However, the enactment of these valued qualities was undermined by the daily struggles of health care seekers and providers to meet their individual needs, necessitating the navigation of strong power relationships within health facilities and the district at large. Those in the weakest position to navigate power had the poorest ability to enact the qualities they aspired to in health care, whether as providers or seekers of care. We argue that achieving aspirations for qualities valued in health care will require a genuine reorientation of focus by health workers and their managers towards patients, through a renewed respect and support for these providers as professionals.

## Abbreviations

FGD: Focus group discussion; HC: Health centre; IQR: Interquartile range; PHC: Primary health care

## Competing interests

The authors declare that they have no competing interests with respect to the research, authorship, and/or publication of this article.

## Authors’ contributions

CIRC and SGS conceived and designed the study. JK, LT, CN and MK undertook data collection and analysis with CIRC. All authors contributed to the interpretation of the data; CIRC drafted the paper and all authors undertook critical revisions of the manuscript and read and approved the final manuscript.

## Authors’ information

CIRC, PhD, is a lecturer in social science in the department of Global Health and Development at the London School of Hygiene & Tropical Medicine, London, United Kingdom.

JK, BA, is a social scientist at the Infectious Diseases Research Collaboration, Makerere University College of Health Sciences, Kampala, Uganda.

LT, BUP, is a social scientist at the Infectious Diseases Research Collaboration, Makerere University, Kampala, Uganda.

CN, BCP, is a social scientist at the Infectious Diseases Research Collaboration, Makerere University College of Health Sciences, Kampala, Uganda.

MK, BSWSA, is a social scientist at the Infectious Diseases Research Collaboration, Makerere University College of Health Sciences, Kampala, Uganda.

DD, MSc, is a research fellow in the Department of Clinical Research at the School of Hygiene & Tropical Medicine, London, United Kingdom.

SGS, MD, PhD, is a clinical senior lecturer in the Department of Clinical Research at the London School of Hygiene & Tropical Medicine, London, United Kingdom.
